# Outside-Host Growth of Pathogens Attenuates Epidemiological Outbreaks

**DOI:** 10.1371/journal.pone.0050158

**Published:** 2012-11-30

**Authors:** Ilona Merikanto, Jouni Laakso, Veijo Kaitala

**Affiliations:** 1 Department of Biosciences, University of Helsinki, Helsinki, Finland; 2 Department of Mental Health and Substance Abuse Services, National Institute for Health and Welfare, Helsinki, Finland; 3 Centre of Excellence in Biological Interactions, Department of Biological and Environmental Science, University of Jyväskylä, Jyväskylä, Finland; Wadsworth Center, United States of America

## Abstract

Opportunist saprotrophic pathogens differ from obligatory pathogens due to their capability in host-independent growth in environmental reservoirs. Thus, the outside-host environment potentially influences host-pathogen dynamics. Despite the socio-economical importance of these pathogens, theory on their dynamics is practically missing. We analyzed a novel epidemiological model that couples outside-host density-dependent growth to host-pathogen dynamics. Parameterization was based on columnaris disease, a major hazard in fresh water fish farms caused by saprotrophic *Flavobacterium columnare*. Stability analysis and numerical simulations revealed that the outside-host growth maintains high proportion of infected individuals, and under some conditions can drive host extinct. The model can show stable or cyclic dynamics, and the outside-host growth regulates the frequency and intensity of outbreaks. This result emerges because the density-dependence stabilizes dynamics. Our analysis demonstrates that coupling of outside-host growth and traditional host-pathogen dynamics has profound influence on disease prevalence and dynamics. This also has implications on the control of these diseases.

## Introduction

Many pathogens are able to survive and replicate in the environment outside-host, e.g., via saprotrophism [1], [2]. These kinds of pathogens can also be called opportunists as the host specificity is often low and growth within host is only an alternative reproductive strategy. The key in the transmission and survival of opportunist pathogens is that they can delay their extinction or survive indefinitely in the outside-host environment. Therefore the opportunists may thrive even though all susceptible hosts would either be treated or removed. In contrast, obligatory pathogens cannot replicate in the outside-host environment and often have higher host specificity. Thus, disease dynamics is likely to differ between opportunist and obligatory pathogens. Opportunist pathogens with the capacity of growing outside-host are also plausible ancestors in the evolution of obligatory pathogens [1]. It has also been suggested that selection favors an opportunistic strategy in general [3], [4].

Although not often recognized, opportunist pathogens are very common and present a significant economical burden and health risk, yet the ecological and evolutionary dynamics of these organisms is poorly understood. Opportunist pathogens in humans include, e.g., cholera (*Vibrio cholera*) and lung infections (*Pseudomonas aeruginosa* and *Legionella pneumophila* [5]–[8]. Cholera outbreaks are common in countries where sanitation and drinking water quality are poor [9], [10]. Lung inflammations on the other hand pose a lethal treat globally to patients with compromised immunity [11]. Other examples of opportunist pathogens are for instance bacteria from *Listeria* and *Flavobacterium* genus, such as *L. monocytogenes, F. psychrophilum* and *F. columnare* [12]–[15]. In particular, fish columnaris disease caused by *F. columnare* has become a major problem in fresh water fish farms cultivating salmonids in Finland and channel catfish (*Ictalurus punctatus*) in the United States [14]–[16].

*F. columnare* has the potential to survive through saprotrophism in the outside-host environment indefinitely and causes opportunistically infections as susceptible host are present [14]–[15]. Infection can result in the death of an entire fish population in a cultivation tank [17]. Also *F. psychrophilum* causes severe fish diseases, Cold-Water Disease and Rainbow Trout Fry Syndrome, in fish farms [12].

To our knowledge, only few models acknowledging outside-host growth has been reported, and the models mostly consider only short-term processes [18], [19]. Theory on long-term disease dynamics of opportunist pathogens does not yet exist. Few theoretical models have been developed for environmentally transmitted pathogens that are able to survive outside the host for a certain time period [20]–[23], but these models are not suitable for opportunist pathogens that can interact with other species and replicate in a density-dependent manner in the outside-host environment. Many previous models regarding host-parasite interaction often assume a trade-off between virulence and transmission. High virulence would eventually increase mortality of the hosts and therefore weaken pathogen growth, as there are less susceptible hosts present. As a consequence, host dependent obligatory pathogen would die out when the host population density becomes too low [24]–[27]. There are host-dependent mechanisms that can enable evolution of high virulence, such as short-sighted within-host strain competition [25]–[33]. The concept of outside-host growth of an opportunist pathogen offers another, novel pathway to high virulence: as the fitness of an opportunist pathogen on is partially independent of the host, the trade-off between virulence and pathogen growth can be weakened or even removed altogether. The ability to survive and replicate in the outside-host environment could therefore promote high-enough virulence that leads to host extinction. Given these obvious discrepancies between the assumptions of the traditional theory of disease dynamics, and the properties of opportunist pathogens, it is essential to further the theory on the dynamics of opportunist diseases. Opportunist disease model can also be to some extent compared to predator-prey systems with more than one prey species. Theoretical work in these systems is also sparse at the moment [34].

Here we introduce a novel model that couples density-dependent growth in the environment to host-pathogen dynamics and analyze the long-term dynamics of the system. Parametrization of the model analyses are based on fish columnaris disease. We demonstrate that the ability to replicate in the outside-host environment can under some conditions lead to host extinction but not necessarily to the extinction of the opportunist pathogen. The model can also produce stable or cyclic dynamics (outbreaks), where the pathogen growth in the outside-host environment regulates the frequency and intensity of outbreaks. Especially, the outside-host growth seems to be source for unstable dynamics and increasing the strength of its density dependence has stabilizing effect on the host-pathogen dynamics in a wide range of the parameter space. Growth in the environment outside-host therefore has a profound influence on disease prevalence and dynamics that differ from the traditional theory of host-pathogen dynamics.

## Methods

### Model of opportunist pathogen-host interaction

We consider a deterministic continuous time model for opportunist pathogen-host interaction. The model combines SI dynamics based on model G of Anderson & May (1981) and outside-host growth of the pathogen to describe changes in time (*t*) in the densities of susceptible hosts (*S*), infected hosts (*I*) and pathogens in the environment outside-host (*P*):
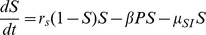
(1)

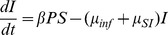
(2)

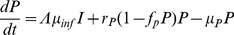
(3)


Susceptible host population (eqn. 1) grows logistically with a growth rate *r_S_* in a density-dependent way (host carrying capacity is assumed equal to 1). Susceptible hosts die at a rate *μ_SI_* and are infected at a rate *β*. Infected host population (eqn. 2) increases depending on transmission rate of infection (*βS_t_P*_t_). We assume that infected hosts are unable to replicate. We also assume that that infected hosts are not competing for resources with susceptible hosts. Density of *I* decreases by death to infection (*μ_inf_*) or due to other causes (*μ_SI_*). *μ_inf_* is used to measure virulence. Equation (3) describes density change in the pathogen population outside-host (*P*) in time (*t*). Pathogen population outside-host (eqn. 3) increases depending on release rate (*Λ*) of new pathogen as infected hosts die due to infection (*μ_inf_*). *P* increases logistically due to opportunist growth rate (*r_P_*) in the outside-host environment, where *f_P_* describes the strength of density dependence. Density of *P* decreases due to a density independent death rate *μ_P_*. The effect of opportunist growth on disease dynamics was studied analytically by linearizing eqns. (1–3) around the equilibrium (equilibrium population densities were restricted to be positive). Linearized population dynamics are given in Appendix S1. The Jacobian eigenvalues were investigated for local stability properties [35].

### Parametrization of the model and numerical simulations

Stability of *SIP* community dynamics was studied by using different combinations of parameter values between 1) pathogen growth rates (*r_P_*) and susceptible hosts growth rates (*r_S_*), 2) pathogen growth rates (*r_P_*) and virulence (*μ_inf_*), 3) pathogen growth rates (*r_P_*) and pathogen death rate (*μ_P_*) or 4) pathogen growth rates (*r_P_*) and pathogen release rate (*Λ*). Parameter values were selected to cover a large range of plausible biological values for different host and opportunist pathogen organisms, such as *Flavobacterium* and *Serratia* genus, where many bacterium species are saprotrophic pathogens with multiple potential hosts [36]–[39]. Natural or experimental growth and mortality values due to infection regarding *Flavobacterium columnare* and *Serratia marcescens* and some of their hosts are given in Table 1. Pathogen growth rates were assumed to be lower in the analyses than those measured in experimental studies to represent more realistic situation found in natural habitats. In nature, hosts can have higher mortality to infection than to other causes [20]. Mortality of the hosts due to other reasons than to infection (*μ_SI_*) was therefore standardized to a low value. Pathogen mortality (*μ_P_*) day^−1^ was varied corresponding to realistic mortality values measured in bacteria. For example aquatic bacteria have been measured mortality rates between 0.01–0.03 h^−1^ [40]. The transmission rate (*β*) for the pathogen was kept low, because infectiveness is by definition lower in opportunist pathogens as compared to obligatory [41]. Parameter values used in the analyses are given in Table 2.

**Table 1:** Reproduction (*r*) and mortality values due to infection (*μ_inf_*) for saprotrophic pathogens *Flavobacterium columnare* and *Serratia marcescens* and some of their hosts based on experimental studies.

*Pathogen*	*r_P_*	*Host*	*μ_inf_*	*r_S_*
*F. columnare*	2.4–7.2 day^−1^ [39]	Atlantic salmon, *Salmo salar*	0.2–0.3 day^−1^ [50]	0.2–1.7 day^−1^ [52]
		Rainbow trout, *Oncorhynchus mykiss*	0.2–0.4 day^−1^ [50]	0.08–0.4 day^−1^ [53], [54]
		Brown trout, *Salmo trutta*	0.01–0.05 day^−1^ [50]	0.05–0.34 day^−1^ [55], [56]
		Chinook salmon, *Oncorhynchus tshawytscha*	0.01–0.05 day^−1^ [50]	0.2–4.4 day^−1^ [57]
		Arctic charr, S*alvelinus alpinus*	0.2–0.3 day^−1^ [50]	0.2–9.1 day^−1^ [58], [59]
		Channel catfish, *Ictalurus punctatus*	0.01–1.0 day^−1^ [38]	2.3–19.7 day^−1^ [60]
		Zebra fish, *Danio rerio*	0.2–0.4 day^−1^ [51]	15–200 day^−1^ [61]
*S. marcescens*	2.4–6 day^−1^ [62]	*Drosophila melanogaster*	0.002–1.0 day^−1^ [63]	11–41 day^−1^ [64]

In the fish host reproduction rates, the number of eggs produced per kg during a year, average weight range of mature fish and survival rate of eggs to fry has been taken into account.

**Table 2:** Parameter values that were used in stability analysis.

*Parameter*	*Explanation of the parameter*	*Parameter values*	*Exceptions in parameter values*
*r_S_*	Susceptible host growth rate	0.01	0.001–0.5 (Fig. 1D, 3D, 4D)
*r_P_*	Pathogen growth rate outside-host	0.001–0.5	0.06 (Fig. 2A) 0–0.15 (Fig. 2B)
*μ_SI_*	Mortality of the susceptible and infected hosts due to other reasons than infection	10^−3^	
*μ_inf_*	Virulence (Mortality of the infected hosts due to infection)	0.1	0.001–0.5 (Fig. 1A, 3A, 4A)
*μ_P_*	Pathogen mortality outside-host	0.1	0.1–0.5 (Fig. 1B, 3B, 4B)
*β*	Pathogen transmission rate to susceptible hosts from environment	10^−5^	
*Λ*	Pathogen release rate from infected hosts when they die	10^5^	5×10^3^–1.5×10^5^ (Fig. 1C, 3C, 4C)
*f_P_*	Negative influence of pathogen population density on its growth	10^−5^	0–10^−6^ (Fig. 2A)

We carried out four different stability analyses where two parameters were varied at a time. One parameter was always the environmental growth rate of the pathogen (*r_P_*). The parameters were given 100 different values from the value range used. For the resulting 100^2^ combinations, the *SIP* community dynamics were simulated for 1700 days.

We simulated the model (1)–(3) for 3500 days to record attributes of the *SIP* community dynamics. Bifurcation diagrams were obtained by scoring the minimum and maximum values of population fluctuations after removing the initial transient. The numerical analysis of the model was performed with MATLAB v. 2011b (ODE45 solver, default tolerance settings).

## Results

As a starting point it is worth of considering a variant of SI model (1)–(2) where the inflow of pathogens, *P*, is assumed to be constant. A straightforward analysis provides a necessary and sufficient condition for positive equilibrium *S*>0 and *I*>0: 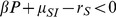
(4)


The eigenvalues of the two-dimensional system are 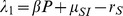 and 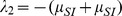. Thus, both eigenvalues are strictly negative and the positive equilibrium is locally stable whenever it exists. In conclusion, any instability that occurs in the SIP model (1)–(3) is caused by the dynamics of the pathogen.

Consider next the pathogen dynamics (3) in the absence of density dependent growth, that is r_P = 0. A straightforward application of the results of Figures S1 and S2 provides us the first eigenvalue: 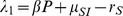


which according to (4) is negative. The two other eigenvalues λ_2,3_ satisfy the eigenvalue equation (35):



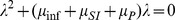
.

Thus, λ_2_ is real and negative and λ_3_ = 0. Consequently, the system is now marginally stable. It follows that the possible genuine instabilities in the host-pathogen system (1)–(3) are due to the density dependent growth of the pathogen in the environment. When the density dependent growth is involved in the system then the analyses of the system becomes much more complicated as all the parameters of the system are involved in driving the behaviour of the system in a strongly nonlinear way. Moreover, as it appears our analysis below many parameters have joint consequences on the dynamics of the system. Hence, we next rely on simulation studies in characterizing the dynamics of the system with opportunistic pathogen.

**Figure 1:**
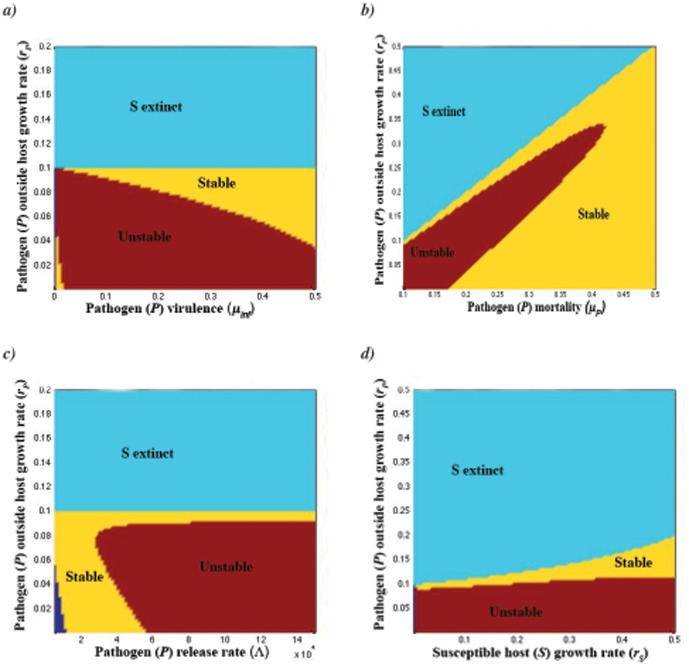
Stability of the *SIP* community dynamics in different combinations of outside-host growth rate of pathogen (*r_P_*) parameter values and parameter values of a) virulence (*μ_inf_*), b) pathogen mortality outside-host (*μ_P_*), c) release rate (*Λ*) and d) susceptible host growth rate (*r_S_*). Dark blue: Pathogen population outside-host (*P*) goes to extinction. Light blue: Susceptible host population (*S*) goes extinct. Yellow: *SIP* community dynamics are locally stable. Red: *SIP* community dynamics are locally unstable. Used parameter values are shown in Table 2.

**Figure 2:**
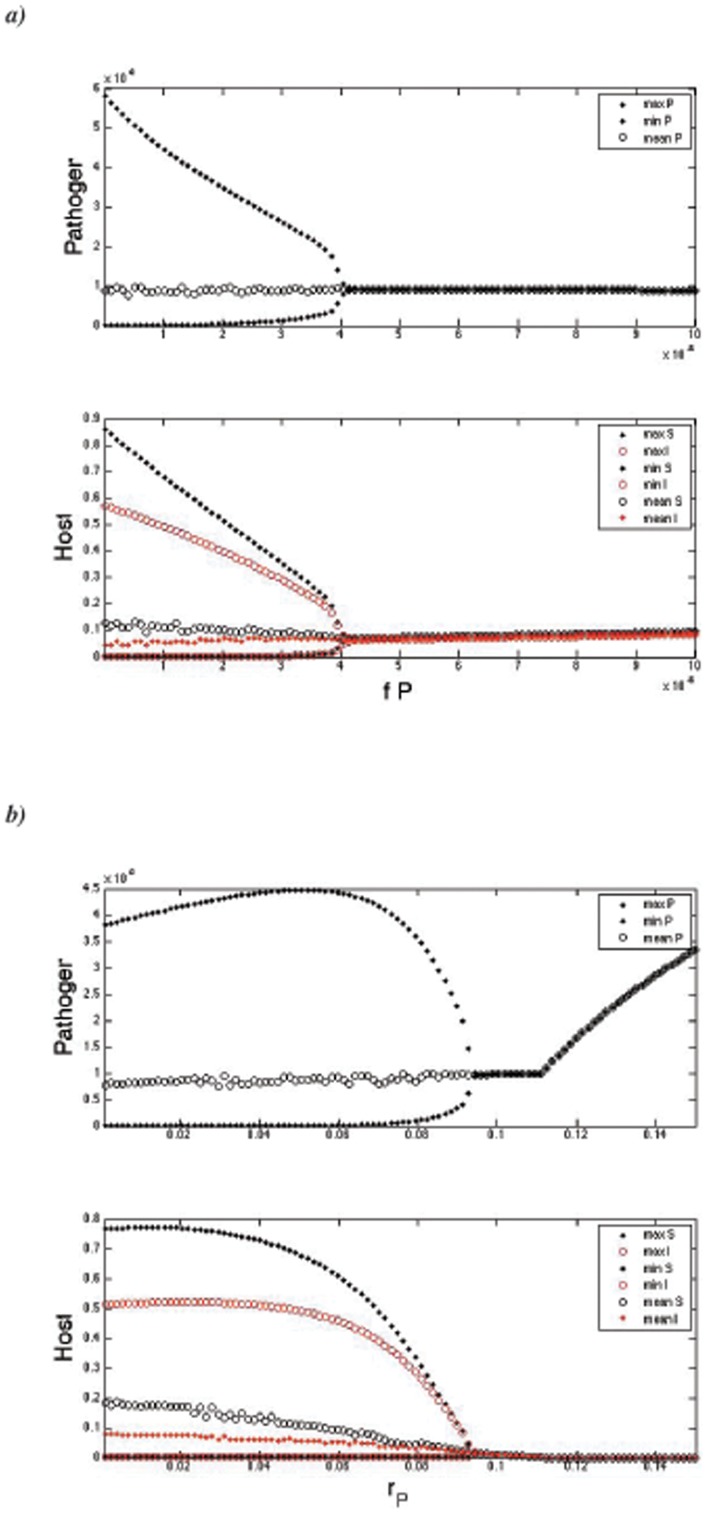
Stabilizing effect of pathogen density-dependent growth in the outside-host environment. Bifurcation diagrammes indicate mean densities and the minimum and maximum values in the population fluctuations of the pathogen and host after the initial transients in the simulations have been removed. a) Increasing the strength of density-dependence stabilizes the population dynamics at *f_P_* = 4.2×10^−5^ (*r_P_* = 0.06). b) For increasing pathogen growth rate population dynamics stabilize at *r_P_* = 0.096 while host extinction occurs at *r_P_* = 0.112. Used parameter values are shown in Table 2.

Increased pathogen virulence *μ_inf_* (Figure 1A), pathogen mortality *μ_P_* (Figure 1B), and the strength of density-dependence *f_P_* (Figure 2A) have a stabilizing effect on the *SIP* system. As *f_P_* increases, susceptible hosts do not have enough time to reach higher maximum densities (Figure 2A). By plotting time series, this seems to be because if density dependence increases, the period of population fluctuations shortens. In contrast, increasing pathogen release rate from the host (*Λ*) destabilizes system dynamics (Figure 1C). Also, increased growth of the pathogen (*r_P_*) in the outside-host environment was destabilizing, given that *r_P_* close to *μ_P_* (Figure 1D, 2B). This happens also when *r_P_* is above *μ_P_*, given that the susceptible host growth rate (*r_S_*) is allowed increase (Figure 1). However, depending on *r_S_*, *Λ* and *μ_P_*, also a sequence of stable to periodic to stable to extinct dynamics can occur when *r_P_* increases (Figure 1B–D). The host population (*S+I*) becomes often extinct when pathogen growth rate exceeds its natural mortality, i.e., *r_P_>μ_P_*, depending on susceptible host growth rate *r_S_* (Figure 1A–D, 2B). In the absence of both the pathogen's ability to have net growth outside the host (i.e., *r_P_<μ_P_*) and the benefit from inside host growth (i.e., when *r_S_* , *μ_inf_* and *Λ* are very low), pathogen population outside host (*P*) goes extinct (Figure 1A, 1C, 1D).

The equilibrium density of pathogen *P* increases with both increasing *r_S_* (Figure S1D) and *r_P_* (Figures S1A, S1B, S1C). The equilibrium density of *P* is maximized when mortality to infection (*μ_inf_*) or release from the host (*Λ*) is low (Figures S1A and S1C). In situations where host (*S*) goes extinct, the pathogen (*P*) necessarily does not, due to the net environmental growth (*r_P_* is close to zero) (Figure S1A–C, Figure S2A–C).

## Discussion

We studied a new class of epidemiological models, where we assume density-dependent growth of a pathogen in the outside-host environment. Traditional epidemiological models on the other hand assume that pathogens do not actively grow in the outside-host environment, for which reason they poorly describe disease dynamics of opportunistic pathogens, such as *Vibrio cholera, Pseudomonas aeruginosa, Flavobacterium columnare, F. psychrophilum* etc. We found that density-dependent growth in the outside-host environment generates disease dynamics that can strongly differ from the traditional SI-models. Importantly, density-dependent growth in the outside-host environment has a strong stabilizing effect on the host-pathogen system. Outside-host replication also enables opportunist pathogens to remain in the environment when the density of susceptible hosts is too low for host-dependent persistence, or in the absence of the hosts. Thus, outside-host growth and the associated density depence is a potential ecological mechanism that can regulate disease outbreaks.

The idea that pathogens are able to survive in the outside-host environment is not new. For example, Day (2002) developed a model where pathogens were able to survive in the outside-host environment by producing resting spores. This model, as well as models by Roche and co-authors (2011) and Boldin & Kisdi (2011), however do not assume that a pathogen can replicate in the outside-host environment. The model developed by Godfray and co-authors (1999) acknowledged outside-host growth of a pathogen and competition in the outside-host environment. However, this model assumes density-independent outside-host growth, and thus, it applies only to processes with limited time horizon, e.g., short-term biological control [18]. To our knowledge, only one previous study considers density-dependent growth outside-host [19]; Joh and co-authors however focused on outbreak thresholds. Their model also lacks background mortality of the pathogen in an environmental reservoir, and thus is not realistic for modelling long-term dynamics of opportunist disease.

Our model can produce cyclic dynamics (outbreaks) where the pathogen growth in the outside host environment regulates the frequency and intensity of outbreaks. In the traditional SI-models destabilizing factors of host-pathogen dynamics include low reproduction rate of susceptible hosts, time lag between pathogen replication inside host, and transmission to new hosts [42]. However, in our model increase in the outside-host pathogen growth rate, and release rate of new pathogens promote cyclic dynamics (outbreaks). The size of the free-living pathogen population (directly related to the increase of new infections) is increased either by higher pathogen release rates, higher growth rate of susceptible hosts, or trivially by higher pathogen growth rates in the outside-host environment.

Also increasing the growth rate of susceptible host promotes cyclic dynamics, when the pathogen growth in the outside host is near pathogen mortality. However, as the pathogen growth in the outside host increases, higher growth rate of susceptible host promotes stable dynamics. As the pathogen growth in the outside host increases even further, the result is host extinction. It is possible to make some comparisons between opportunist disease dynamics and predator-prey dynamics. In predator-prey interactions, unstable dynamics are possible when prey population is abundant and the growth rate of predator is on the other hand low [34], [43].

Opportunist pathogenicity functions as a density amplifier of otherwise saprotrophic bacteria growth as opportunists can often replicate more efficiently via pathogenicity than via saprotrophism due to host being richer resource as compered to saprotrophic resource. Abrams and Matsuda (1996) predicted that there might not be apparent competition between prey species if efficient predation of other prey species increases death rate of both prey species similarly [44]. We assume that opportunist pathogenicity leads to trade-off with the efficiency to grow saprotrophically, but as opportunists are able to increase their abundance in greater quantities by pathogenicity in the outside host environment, leads this also to greater consumption of saprotrophic resources than if bacteria would only grow saprotrophically. However, as outside-host growth might lead to host extinction, this situation could be compared to apparent competition found in predator-prey systems [45].

It is notable that the outside-host density-dependence and increased pathogen mortality stabilize host-pathogen dynamics. In traditional SI-models, the replication of pathogens inside hosts [46] functions as a density-dependent constrain that also has a stabilizing effect that could be equivalent to density-dependent constrains in the outside-host environment. Increased density-dependency and higher pathogen mortality outside hosts are likely to be stabilizing because the pathogen has a weaker regulatory effect on susceptible hosts as pathogen growth is more restricted. Also, in our model high virulence stabilizes host-pathogen dynamics similarly to the traditional SI-models. Higher virulence increases mortality of infected hosts and therefore speeds pathogen release to outside host environment. This strengthens intraspecific competition for resources in the outside-host environment. High virulence therefore functions indirectly as a density-dependent mechanism through replication in the outside-host environment.

If we would disregard density dependency in our model and assume that pathogen density would equilibrate faster than host densities, the model could be 2-dimensional similarly as Lotka-Volterra predator-prey interaction, where change in pathogen population density in time (eq. 3) could be solved in terms of infected host population density (eq. 2). This kind of model would give linear functional response with stable equilibrium dynamics.

The fact that the opportunist pathogen does not necessarily need the host for long-term survival could lead to development of high virulence, which is in contrast with predictions made for obligatory pathogens where virulence is expected to decline in over time [24]–[27]. High host infection rate coupled to high pathogen release rate increases the pathogen fitness. This could offer a competitive benefit to opportunist pathogens, e.g., when different microbe strains are competing for the same resources in the outside-host environment. Outside-host growth also makes disease outbreaks possible when the density of susceptible hosts increases after an epidemic. For example, *Vibrio cholerae* disease outbreaks are connected to the ability to reproduce outside the hosts in aquatic environment [8].

Our model predicts that opportunist disease outbreaks may occur when density dependent ecological interactions, such as outside-host competition, are relaxed. Intensive plant or livestock farming typically fulfills these conditions. Thus the model could predict how decreasing pathogen growth rate in the outside-host environment could prevent disease outbreaks. Furthermore, other biological control applications are possible. For instance, it has been suggested that thorn-inhabiting bacteria, such as the *Clostridium* genus, have a potential anti-herbivory role in thorny plants [47]. Thus, the model could for instance also be applied to biological control against herbivores by increasing saprotrophic growth of opportunist herbivore pathogens in the outside host environment.

It has been proposed that host-specific enemy, such as a parasite or predator, is often ineffective way to prevent pest populations growth to high densities. Enemy with an alternative food resource on the other hand would be more efficient in biological control, as it is able to sustain high population density by using alternative food resource even if pest population sizes would fluctuate [48]. Opportunist pathogen would in this sense be ideal in biological control as it is able to replicate in the outside host environment independently of the host but prevents efficiently host population growth. For example, saprotrophic *Serratia entomophila* bacteria has been used with success in the biological control of New Zealand grass grub (*Costelytra zealandica*) [18].

The model could also be applied to biological control of the saprotrophically transmitting *Flavobacterium columnare* bacterium. Biological control of *F. columnare*, as well as for some other fish pathogen bacteria from the *Flavobacterium* genus, is needed due to the negative side effects of increasing use of antibiotics. It has been suggested that conditions in fish tanks are ideal environment for the evolution of high virulence opportunist bacteria and therefore opportunist disease outbreaks. These conditions include high susceptible host density, lack of natural bacterial predators or competitors of *F. columnare*, and high availability of dead fish material, fish food [15]. The antibiotic treatment of infected fish is unable to remove *F. columnare* from the fish tanks, as they are able to survive and replicate outside fish [14], and are constantly reintroduced to the tanks from inflow water from natural fresh waters [49]. We suggest that the more effective way to treat columnaris disease in fish farm would be more efficient removal of saprotrophic resources, such as dead fish material and faeces from the tanks and thus decrease pathogens' ability to grow outside the host and by increasing the diversity of natural bacterial enemies in fish tanks.

## Summary

We modeled the dynamics of opportunist disease capable of density-dependent environmental growth. The disease cycles (outbreaks) crucially depend on the outside-host density-dependent growth. Interestingly, the density dependent outside-host pathogen growth strongly stabilizes disease dynamics. Saprotrophic opportunism is an efficient life-history strategy because the ability to replicate in the outside-host environment potentially gives large fitness benefit as compared to non-pathogenic strains or obligatory pathogen. That the opportunist pathogens are also able to survive in the outside-host environment even when there are no susceptible hosts available, could promote the evolution of higher virulence regardless of the virulence-transmission tradeoff that the obligatory pathogens have to face. Capability to the outside-host growth is also a novel ecological mechanism for disease outbreaks.

## Supporting Information

Appendix S1 **Local Stability Analysis.**
(DOC)

Figure S1 **Equilibrium densities of pathogen population outside-host (** ***P*** **) with different combinations of outside-host growth rate of pathogen (** ***r_P_*** **) values and parameter values of a) virulence (** ***μ_inf_*** **), b) pathogen mortality outside-host (** ***μ_P_*** **), c) release rate (** ***Λ*** **) and d) susceptible host growth rate (** ***r_S_*** **).** Used parameter values are shown in Table 2.
(EPS)

Figure S2 **Equilibrium densities of susceptible host population (** ***S*** **) with different combinations of outside-host growth rate of pathogen (** ***r_P_*** **) values and parameter values of a) virulence (** ***μ_inf_*** **), b) pathogen mortality outside-host (** ***μ_P_*** **), c) release rate (** ***Λ*** **) and d) susceptible host growth rate (** ***r_S_*** **).** Used parameter values are shown in Table 2.
(EPS)
