# Supplementary material for: Outside-Host Growth of Pathogens Attenuates Epidemiological Outbreaks
Source: PLoS One. 2012 Nov 30;7(11):e50158. doi: 10.1371/journal.pone.0050158 (PMC3511454; doi:10.1371/journal.pone.0050158)
Supplement: Appendix S1 — Local Stability Analysis. (DOC) [file pone.0050158.s001.doc]

The equilibrium population densities in the *SIP* system (1)-(3) are given as follows:

$$\begin{matrix} \\ \overline{S}=\frac{\left[(-r_{P}+\mu_{P})\beta+(r_{P}f_{P})(\mu_{SI}+r_{S})\right](\mu_{\inf}+\mu_{SI})}{\Lambda\mu_{\inf}\beta^{2}+r_{S}\left[(\mu_{\inf}+\mu_{SI})(r_{P}f_{P})\right]} \end{matrix}$$

$$\overline{P}=(r_{S}\left[1-\overline{S}\right]-\mu_{SI})/\beta$$

$$\begin{matrix} \\ \overline{I}=\beta\overline{S}\overline{P}/(\mu_{\inf}+\mu_{SI}) \end{matrix}$$

Population dynamics linearized at equilibrium become

$$\begin{matrix} \frac{ds}{dt}=r_{S}\left[1-2\overline{S}\right]s-\beta\overline{P} s-\mu_{SI}s-\beta\overline{S} p \\ \frac{di}{dt}=\beta\overline{P} s-(\mu_{\inf}+\mu_{SI})i+\beta\overline{S} p \\ \frac{dp}{dt}=\Lambda\mu_{\inf}i+r_{P}\left[1-2f_{P}\overline{P}\right]p-\mu_{P}p \end{matrix}$$

The Jacobian matrix for the system is given as

$$A=\left[\begin{matrix} r_{S}\left(1-2\overline{S}\right)-\beta\overline{P}-\mu_{SI} & 0 & -\beta\overline{S} \\ \beta\overline{P} & -(\mu_{\inf}+\mu_{SI}) & \beta\overline{S} \\ 0 & \Lambda\mu_{\inf} & r_{P}\left(1-2f_{P}\overline{P}\right)-\mu_{P} \end{matrix}\right]$$

*SIP* community dynamics are locally stable if all the eigenvalues of the Jacobian matrix have negative real part (35).
